# North-Eastern Hospital for Children

**Published:** 1904-06-25

**Authors:** Amherst of Hackney, Susan Somerset, William Cecil, M. Rüffer

**Affiliations:** President.; Vice-President.; Chairman.; Treasurer.


### EDITOR'S LETTER-BOX.

[Our Correspondents are reminded that prolixity is a great bar to publication, and that brevity of style and conciseness of statement greatly facilitate early insertion.]

### NORTH-EASTERN HOSPITAL FOR CHILDREN.

*To the Editor of* THE HOSPITAL.

June 21, 1904.

SIR,—A new building, providing 57 additional beds and much other urgently-needed accommodation, has recently been added to the North-Eastern Hospital for Children, Hackney Road, Bethnal Green, at a cost (including freehold land) of £44,000, towards which a sum of £35,600 has so far been given.

Her Royal Highness Princess Louise, Duchess of Argyll, has graciously consented to declare this building open on July 13, and will then receive purses of £5 5s. and upwards in aid of the funds, together with a list of other sums which it is hoped will be contributed.

The new wards were completed in September 1903, and, in view of the urgent demands of the surrounding crowded districts, were at once brought into use, being filled within a few days. Notwithstanding that the available beds were thus increased to 114, the pressure on the In-Patient accommodation has continued to be severe, and the Hospital may be said to be always full. The Out-Patients number 1,200 per week, including 115 accident and emergency cases.

The expenditure having been thus increased from £6,500 to £11,000 a year, the Hospital is now in great need of immediate assistance for the maintenance of this important work, and we earnestly plead for donations and new annual subscriptions to the amount of £5,000 to tide over the present serious difficulties.

If we were also fortunate enough to obtain £9,000 to pay off the building fund debt and £25,000 to complete the scheme of enlargement, we should be very grateful; but circumstances compel us to appeal in the first place for funds for the maintenance of the Hospital, and we trust that we shall not ask in vain for so good a work.

It should be noted that King Edward's Hospital Fund annually subscribes £500 for general expenses, and has, in addition, given £3,250 towards the building fund.

Cheques, etc., crossed "Barclay and Company, Limited," should be sent to the Treasurer at the Hospital, and the smallest contribution will be thankfully received.

We are, Sir, your most obedient servants,

AMHERST OF HACKNEY, President.
SUSAN SOMERSET, Vice-President.
WILLIAM CECIL, Chairman.
M. RÜFFER, Treasurer.

